# Feeding the estrobolome with sweet prebiotics?

**DOI:** 10.3389/fnut.2026.1957439

**Published:** 2026-09-25

**Authors:** Lorenzo Agoni, Canio Martinelli, Salvatore Cortellino, Antonio Giordano, Francesco De Seta

**Affiliations:** 1 Unit of Obstetrics and Gynecology, Fondazione Poliambulanza Istituto Ospedaliero, Brescia, Italy; 2 Sbarro Institute for Cancer Research and Molecular Medicine and Center of Biotechnology, College of Science and Technology, Temple University, Philadelphia, PA, United States; 3 Unit of Obstetrics and Gynecology, Department of Human Pathology of Adult and Childhood “Gaetano Barresi”, University of Messina, Messina, Italy; 4 Clinical and Translational Oncology, Scuola Superiore Meridionale (SSM), Naples, Italy; 5 Department of Medical Biotechnology, University of Siena, Siena, Italy; 6 Department of Obstetrics and Gynecology, IRCCS San Raffaele Scientific Institute, Milan, Italy

**Keywords:** estrobolome, estrogen, estrogen recirculation, gut microbiome, gut microbiota, sweeteners, women’s health, β-glucuronidases

## Abstract

**Background:**

Sweeteners are widely consumed, yet their effects on the gut microbiota remain incompletely characterized, while their potential influence on the estrobolome has received little direct investigation. Although many sweeteners are absorbed in the small intestine, a variable fraction reaches the colon, where it interacts with the gut microbiota. Some sweeteners may also meet the definition of a prebiotic when they are selectively utilized by host microorganisms and such utilization confers a demonstrated health benefit. Because the estrobolome encompasses microbial genes encoding estrogen-metabolizing enzymes, including β-glucuronidases and sulfatases, sweetener-induced changes in gut microbial composition or function could plausibly influence enterohepatic estrogen metabolism and systemic estrogen availability.

**Methods:**

This narrative review synthesizes current evidence on the effects of different sweeteners on the gut microbiota and examines their potential implications for the estrobolome. We summarize the consensus definition of prebiotics, the classification, metabolism, and colonic availability of major sweeteners, and review the biological basis of the estrobolome, with particular emphasis on microbial estrogen metabolism.

**Results:**

Experimental and human studies indicate that individual sweeteners can modify gut microbial composition and metabolic activity, but the direction and magnitude of these effects vary substantially according to the compound, dose, study model, and host context. However, direct studies evaluating the impact of sweeteners on estrobolome function are lacking, and potential effects currently remain inferential, based largely on reported changes in microbial taxa known or presumed to harbor genes encoding estrogen-metabolizing enzymes rather than on direct measurements of gene expression or enzymatic activity.

**Conclusion:**

Current evidence provides a biological basis for considering sweeteners as potential modulators of the estrobolome, although evidence directly linking individual sweeteners to changes in estrobolome function remains lacking. Well-designed multi-omic clinical studies integrating dietary exposure, gut microbiota composition, estrobolome function, estrogen metabolism, and clinically relevant outcomes are needed to establish the physiological and clinical relevance of this relationship and determine whether it can ultimately inform personalized nutritional strategies.

## 1 Introduction

Sweeteners are now ubiquitous in the modern diet, widely used in beverages, confectionery, snacks, desserts, and so-called “sugar-free” foods, but also present in oral medications, supplements, oral hygiene products, and a variety of other goods. Growing concern about obesity, diabetes, and cardiometabolic disease has contributed to a shift from conventional sugars toward low- and non-caloric alternatives (1). Accordingly, their evaluation has centered largely on the purpose of this substitution—reducing sugar and caloric intake—and on conventional metabolic endpoints such as glycemic control and weight management. The shift toward a One Health approach changes the scientific question that must be asked about this transition. One Health recognizes the interdependence of human health, food systems, and the wider biological and environmental context (2). Within this perspective, a dietary transition cannot be evaluated only through its intended metabolic endpoints; it must also be followed through the biological systems with which the resulting exposure interacts. For sweeteners, this makes the gut microbiome a necessary level of investigation.

By contrast, the effects of these compounds on the gut microbiome have been less thoroughly explored and remain incompletely understood, especially with regard to long-term health consequences. The gut microbiota is strongly influenced by dietary habits, which can modulate microbial composition and function. This complex microbial ecosystem interacts with host metabolism, immunity, and neuro-signaling and may influence metabolic, immune, and brain homeostasis (3–5). Following sweetener exposure through this system therefore requires asking not only whether it alters microbial composition, but which microbial functions might connect any such changes to host physiology. Among the microbial functions through which dietary exposures may have systemic consequences, microbial estrogen metabolism is particularly consequential. A lesser-known but key functional component of the gut microbiome is the estrobolome, defined as the collection of microbial genes encoding enzymes, notably β-glucuronidases and sulfatases, capable of metabolizing estrogens and their conjugates, and by extension the taxa that harbor them. Through the deconjugation of estrogens excreted in bile, estrobolome-associated enzymatic activities can facilitate their reabsorption and thereby contribute to enterohepatic estrogen recirculation and systemic estrogen availability (6). Systemic estrogen availability, in turn, can influence multiple estrogen-responsive tissues, including breast, endometrium, bone, the cardiovascular system, and the vaginal mucosa. Emerging evidence links altered estrobolome activity with estrogen-related conditions such as hormone-dependent cancers, metabolic disturbances, and gynecologic disorders (7, 8).

Whether sweeteners could influence this functional system depends in part on their intestinal availability. Their chemical characteristics, absorption, and metabolic fate influence how much of each compound reaches the colon and becomes available to the microbiota. Some sweeteners are fully or largely absorbed in the small intestine, limiting their availability as substrates for colonic microorganisms, whereas others partially escape upper-gut digestion and reach the large intestine, where they encounter the resident microbiota. There, they may be fermented, transformed, or act as xenobiotic signals, potentially altering microbial community composition and metabolic outputs, including short-chain fatty acids (SCFAs) and bile-acid derivatives. These differences mean that sweeteners cannot be treated as a uniform exposure for the colonic microbiota. Although an increasing number of experimental and clinical studies have examined how sweeteners affect the gut microbiome, to our knowledge, none has directly evaluated whether they affect estrobolome function. This is the unresolved point in a dietary transition already occurring at scale: sweetener exposure is widespread, while its potential effects on microbial estrogen metabolism have not been directly examined.

This unresolved relationship is particularly important for women’s health. Estrogen levels and signaling play a central role in reproductive function, bone and cardiovascular health, and the maintenance of a Lactobacillus-dominated vaginal microbiota. Dysregulation of estrogen metabolism has been implicated in conditions such as endometriosis, polycystic ovary syndrome (PCOS), menopausal symptoms, and hormone-dependent cancers (7, 9, 10). If sweetener-induced changes in gut microbial composition or function can influence estrogen-metabolizing genes or enzymatic activities, then sweetener consumption could represent a modifiable factor within the broader network of diet–microbiota–hormone interactions relevant to women’s health. At present, however, this relationship remains inferential, as direct evidence linking sweetener exposure to estrobolome function is lacking.

In this narrative review, we examine this continuum, from the widespread use of sweeteners in modern food systems to their intestinal availability, documented effects on gut microbial communities, and potential implications for the estrobolome. We first summarize key features of the gut microbiome and the biological basis of the estrobolome, with particular emphasis on microbial pathways involved in estrogen metabolism. We then classify sweeteners by source, structure, and metabolic fate, emphasizing which compounds reach the colon and in what proportion and considering the potential prebiotic properties of individual compounds. Building on this foundation, we review experimental and clinical evidence on how individual sweeteners affect gut microbial communities and examine how these findings may inform hypotheses regarding potential estrobolome modulation. By following this sequence, we distinguish what is known about sweetener–microbiome interactions from what can currently only be hypothesized about the estrobolome.

## 2 Materials and methods

This narrative review was developed to examine the potential relationship between natural and artificial sweeteners and the estrobolome. We first searched PubMed using the terms “estrobolome” AND “sweetener,” without restrictions on publication date or article type. No relevant publication directly addressing this relationship was identified. We therefore broadened the literature search to examine the available indirect evidence linking sweetener exposure to the gut microbiome and considered these findings in relation to current knowledge of microbial pathways involved in estrogen metabolism.

PubMed was searched using combinations of the terms “gut microbiome,” “gut microbiota,” and “sweetener.” Recent reviews were preferentially used to map the current evidence concerning the effects of specific sweeteners on gut microbial composition and function, while relevant primary and foundational studies were consulted when needed to examine specific microbiological, metabolic, pharmacokinetic, or mechanistic aspects in greater detail. Evidence from *in vitro* studies, animal models, observational studies, and human interventions was considered according to its relevance to the question addressed.

Reported microbiome effects were then interpreted in relation to bacterial taxa and microbial functions associated with estrogen metabolism. Because changes in taxonomic abundance do not directly demonstrate changes in estrogen-metabolizing enzyme expression or activity, such associations were considered hypothesis-generating rather than evidence of direct estrobolome modulation. Direct evidence was distinguished conceptually from microbiome-based inference and from hypothetical endocrine or clinical consequences throughout the review.

This literature search was intended to support a narrative and mechanistic synthesis rather than an exhaustive or systematic evidence assessment. Accordingly, no formal systematic-review procedures, quantitative evidence synthesis, or risk-of-bias assessment were undertaken. The resulting literature was used to construct the comparative framework presented in Table 1 and to identify current knowledge gaps concerning the potential interaction between sweeteners, the gut microbiome, and microbial estrogen metabolism.

**Table 1:** Physicochemical and prebiotic characteristics of common sweeteners and dietary prebiotics.

Molecule	Chemical class	Source	Calories (kcal/g)	Potency (vs. Su.)^1^	Colonic avail. (%)^2^	Fermentation^3^	References
Saccharin	Sulfonamide	Synthetic	0	~200–500	~5–15	No	(30, 58, 60–62)
Acesulfame-K	Sulfonamide	Synthetic	0	~200	~2–5	No	(63–65)
Cyclamate	Sulfamate	Synthetic	0	~30	~50–70	Yes	(29, 66–70)
Aspartame	Peptide	Synthetic	4	~200	0	No	(1, 58, 65, 71)
Neotame	Peptide	Synthetic	0	~7.000–13.000	~50	ND	(29, 72–74)
Alitame	Peptide	Synthetic	1.4	~2.000	~2–22	ND	(66, 75–77)
Advantame	Peptide	Synthetic	0	~20.000	~77–96	ND	(78–80)
Polydextrose	Polysaccharide	Synthetic	1	~0.15	~95	Yes	(45, 46, 81–84)
Sucralose	Disaccharide	Synthetic	0	~600	>85	No	(30, 58, 61, 85)
Sucrose	Disaccharide	Sugarcane/sugar beet	4	1	0	No	(86–89)
Inulin	Polysaccharide	Chicory root/Jerusalem artichoke	1.5	~0.1		Yes	(90–94)
FOS	Oligosaccharide	Chicory root/sucrose	1.5	~0.3–0.6	~90–96	Yes	(95–98)
GOS	Oligosaccharide	Milk/lactose	1.7	~0.3–0.6	~76	Yes	(99–103)
IMO	Oligosaccharide	Corn starch/tapioca starch	1.5–3.3	~0.5	~10	Yes	(104–106)
XOS	Oligosaccharide	Xylan-rich plant biomass	1.5	~0.25–0.5	~100	Yes	(107–110)
Thaumatin	Protein	Katemfe fruit	0	~2.000–3.000	0	No	(111–115)
Monellin	Protein	Serendipity berries	4	~800–3.000	0	No	(1, 116–118)
Miraculin	Protein	Miracle fruit	4	~5.000^4^	0	No	(119–121)
Brazzein	Protein	Oubli fruit	4	~500–2.000	0	No	(111, 122)
Fructose	Monosaccharide	Corn starch/fruit	4	~1.2–1.5	~5–20	Yes	(88, 123–125)
Glucose	Monosaccharide	Corn starch/wheat	4	~0.7–0.8	0	No	(86, 126, 127)
Allulose	Monosaccharide	Fructose	0.2	~0.7	~20–30	No	(128–130)
Tagatose	Monosaccharide	Galactose/lactose	1.5–3.0	~0.9	~80	Yes	(128, 131–133)
Xylitol	Polyol	Birch wood/corn cobs	2.4	~1	~50	Yes	(134–137)
Sorbitol	Polyol	Glucose/fruits	2.6	~0.6–0.7	~20–35	Yes	(86, 138–141)
Lactitol	Polyol	Lactose	2.0–2.3	~0.3–0.4	~84–100	Yes	(142–144)
Mannitol	Polyol	Fructose/glucose	1.6	~0.5–0.6	~75	Yes	(145–147)
Maltitol	Polyol	Starch	2.1–2.4	~0.75–0.90	~25	Yes	(1, 141, 147–149)
Isomalt	Polyol	Synthetic	2.1–2.4	~0.5	~40	Yes	(141, 147, 150, 151)
Erythritol	Polyol	Glucose	0.2	~0.7	~10	No	(152–157)
Steviol glycosides	Glycoside	Stevia leaves	0	~150–450	100	Yes	(158–162)
Rebaudioside A	Glycoside	Stevia leaves	0	~200–400	100	Yes	(160, 163–165)
Mogrosides	Glycoside	Monk fruit	0	~300–500	100	Yes	(166–168)

^1^Sweetening potency relative to sucrose (sucrose = 1). Values are approximate ranges reported in the literature and may vary according to experimental conditions and formulation. Higher values indicate a stronger perceived sweet taste; ^2^Colonic availability: percentage of the compound reaching the colon. Colonic availability determines the fraction potentially available for direct interaction with the colonic microbiota; however, not all compounds reaching the colon are fermented by resident microorganisms; ^3^Fermentation indicates whether the compound can be metabolized by the intestinal microbiota through fermentative pathways. Fermentation alone does not establish prebiotic status, which requires selective utilization by host microorganisms conferring a health benefit. ND, not demonstrated; ^4^Sweetening effect occurs only at acidic pH; at neutral pH the sweetening effect is negligible.

## 3 The gut microbiome and the estrobolome

### 3.1 Composition and metabolic functions of the gut microbiome and estrobolome

The gut microbiome and the estrobolome form an interconnected metabolic network that influences host nutrition, immunity, and endocrine function. The gut microbiome comprises thousands of bacterial taxa and a rich gene repertoire that collectively perform complex metabolic tasks, including degradation of complex polysaccharides, vitamin synthesis, and biotransformation of xenobiotics. Within this broader community, the estrobolome plays a specific endocrine role by modulating the systemic availability of estrogens via the enterohepatic recirculation (11). The adult gut microbiota is dominated primarily by bacteria from the phyla Firmicutes and Bacteroidetes, with lesser contributions from Actinobacteria, Proteobacteria, and Verrucomicrobia. These communities metabolize complex carbohydrates, resistant starch, and host-derived glycans (e.g., mucin), generating SCFAs (acetate, propionate, butyrate) that fuel colonocytes, regulate immune responses, and influence metabolic pathways. They also synthesize certain vitamins (notably vitamin K and several B vitamins), modulate bile acid availability, and produce a broad range of signaling molecules (indoles, phenolic acids, amines) that interact with host receptors in multiple ways. The composition and metabolic output of the microbiota are shaped by diet, medications, host genetics, and environmental exposures, and are central to the regulation of host–microbe homeostasis (11).

The estrobolome is defined as the collection of gut microbial genes, and by extension the bacterial taxa that harbor them, encoding enzymes capable of metabolizing estrogens and their conjugates. Although β-glucuronidases (GUS) and sulfatases (SULF) are the best-characterized microbial enzymes involved in estrogen deconjugation and enterohepatic recirculation, the enzymatic repertoire associated with the estrobolome extends beyond these pathways. Microbial hydroxysteroid dehydrogenases, including 3β- and 17β-hydroxysteroid dehydrogenases (HSDs), participate in steroid transformations, including the interconversion and metabolism of estrogens, while β-glucosidases have been implicated particularly in the hydrolysis of dietary phytoestrogen glycosides (7, 12). However, evidence for these activities is heterogeneous across enzyme classes and microbial taxa, and their distribution and relative contribution within the gut microbial community remain less well characterized than those of GUS and SULF (7). In the context of enterohepatic estrogen recirculation, GUS and SULF therefore constitute the principal functional focus of the present review. These enzymes deconjugate estrogens that have been glucuronidated or sulfated in the liver and excreted into bile, regenerating free, bioactive estrogens that can be reabsorbed into the bloodstream and distributed to peripheral organs. In this way, the estrobolome acts as a microbial “endocrine organ” that fine-tunes enterohepatic recirculation and influences the size and composition of the systemic estrogen pool (6) (Figure 1).

**Figure 1:**
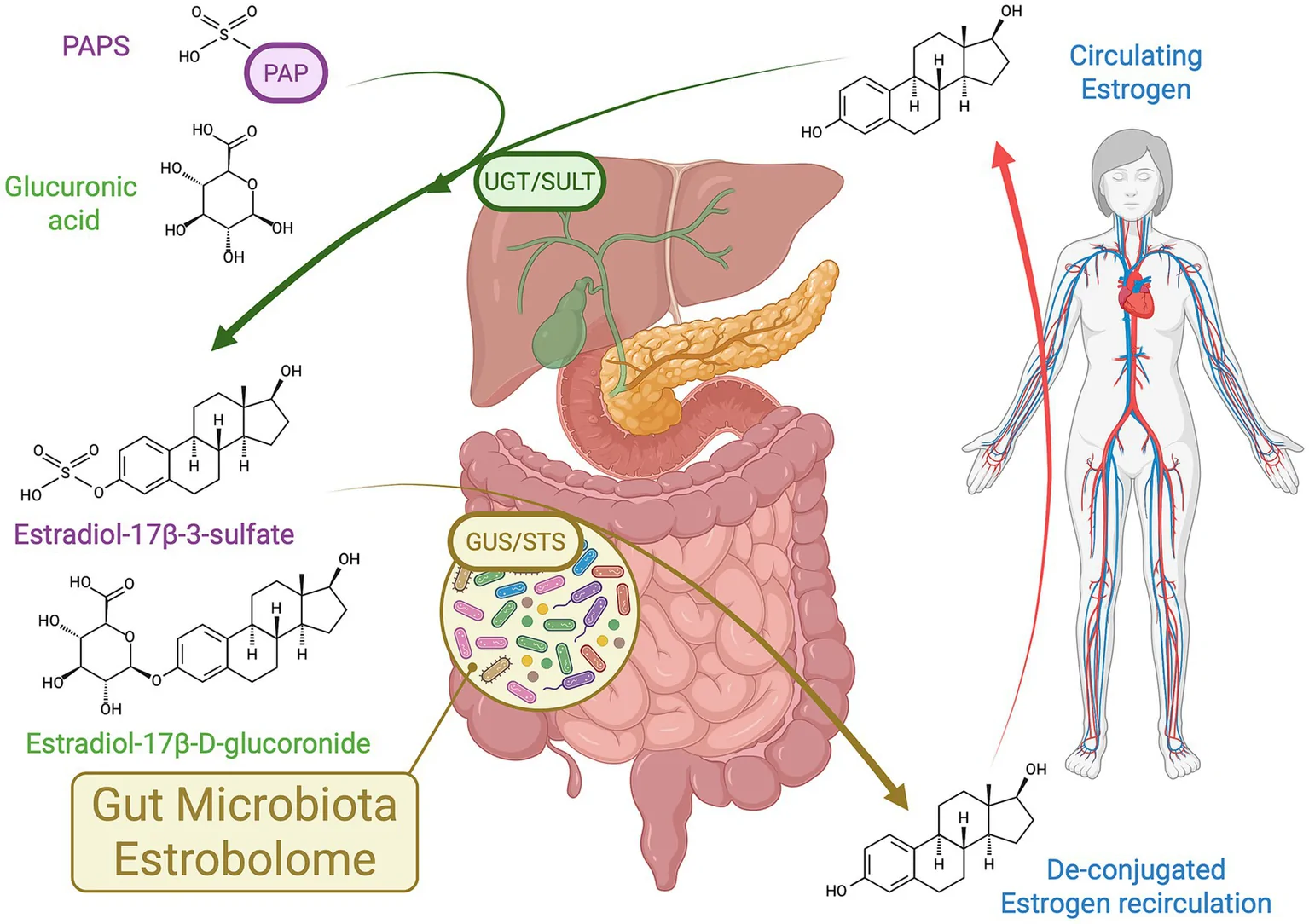
Enterohepatic recirculation of estrogens. Circulating estrogens undergo hepatic conjugation through different pathways, including glucuronidation by UDP-glucuronosyltransferases (UGTs) and sulfation by sulfotransferases (SULTs). In the latter pathway, 3′-phosphoadenosine-5′-phosphosulfate (PAPS) acts as the sulfate donor and is converted to 3′-phosphoadenosine-5′-phosphate (PAP). Conjugated estrogens are subsequently excreted via bile into the intestine, where microbial enzymes associated with the estrobolome, including β-glucuronidases (GUS) and steroid sulfatases (STS), can deconjugate estrogen glucuronides and sulfates, respectively. The resulting free estrogens may then be reabsorbed and returned to the systemic circulation. The figure illustrates 17β-estradiol and its glucuronide and sulfate conjugates as representative examples of these pathways. Created in BioRender.

Key contributors to the GUS- and SULF-mediated estrogen deconjugation include taxa within Bacteroides, Clostridium, Faecalibacterium, Ruminococcus, Escherichia, and Akkermansia, among others. These groups differ substantially in their β-glucuronidase and sulfatase gene repertoires, the cellular localization of the encoded enzymes (intracellular vs. periplasmic or cell-surface associated), and their substrate specificity. For example, some Bacteroides species express periplasmic β-glucuronidases belonging to structural families with relatively broad activity toward small-molecule glucuronides, whereas certain Clostridium and Escherichia strains produce intracellular Loop 1 GUS enzymes with high catalytic efficiency for specific drug or steroid conjugates (13, 14). Faecalibacterium and Ruminococcus species often contribute to the deconjugation of dietary polyphenols and complex glycoconjugates, while *Akkermansia muciniphila* encodes β-glucuronidases that may act at the interface between mucin degradation and steroid metabolism. The abundance, expression, and activity of these enzymes vary markedly between individuals and across time. Importantly, taxonomic abundance should not be regarded as a direct proxy for estrobolome function. The presence or relative abundance of a bacterial genus does not establish the presence, expression, or activity of specific estrogen-metabolizing enzymes, which may vary between species and strains. Consequently, changes in taxa associated with GUS or SULF activity can support biologically plausible hypotheses regarding altered estrogen metabolism but cannot, in the absence of functional measurements, demonstrate changes in estrogen deconjugation or enterohepatic recirculation. They are influenced by dietary patterns (e.g., fiber and fat intake, overall energy load), exposure to antibiotics and other medications, ingestion of xenobiotics (including food additives and sweeteners), intestinal transit time, bile acid composition, and host factors such as age, hormonal status, and underlying disease. Changes in any of these variables can shift the relative contribution of different taxa and enzyme types to overall estrobolome function, potentially altering the balance between estrogen excretion and reactivation and thereby modulating systemic estrogen exposure (15).

### 3.2 Microbial and endocrine consequences of dysbiosis

Dysbiosis refers to qualitative and/or quantitative alterations in the gut microbiota associated with loss of diversity, depletion of beneficial commensals, and expansion of pathobionts or opportunistic organisms. It is often accompanied by decreased butyrate production, altered bile acid metabolism, increased intestinal permeability, and a pro-inflammatory immune tone. Dysbiosis has been linked, variably and not always causally, to a wide range of chronic conditions. In obesity and metabolic syndrome, altered microbial composition and function have been associated with increased energy harvest from the diet, low-grade inflammation, insulin resistance, and dyslipidemia. In inflammatory bowel disease, dysbiosis is typically characterized by reduced diversity, loss of key butyrate-producing commensals, and expansion of pro-inflammatory pathobionts, contributing to epithelial barrier disruption and aberrant immune activation (16). In irritable bowel syndrome, shifts in microbial communities and metabolite profiles (e.g., SCFAs, bile acids, gases) are thought to influence visceral sensitivity, motility, and gut–brain signaling (17). Dysbiosis has also been implicated in autoimmune and allergic diseases, where perturbations in early-life and adult microbiota may skew immune tolerance, promote Th1/Th2/Th17 imbalance, and alter regulatory T-cell function (18). In neuropsychiatric conditions, including depression, anxiety, and autism spectrum disorders, altered gut microbial composition and metabolites have been associated with changes in neurotransmitter pathways, neuroinflammation, and hypothalamic–pituitary–adrenal axis regulation (19). From an endocrine perspective, dysbiosis can modify SCFA profiles, bile acid pools, and microbial enzyme expression, thereby influencing hormone metabolism, including that of estrogens (20).

Estrobolome dysbiosis can be conceptualized as an imbalance in the distribution or functional activity of microbial estrogen-metabolizing pathways. This may involve changes in the abundance of taxa carrying β-glucuronidase or sulfatase genes, altered expression or activity of these enzymes, or shifts in the representation of bacterial strains harboring enzyme variants with different catalytic properties. Such alterations can shift the equilibrium between estrogen excretion and reactivation, leading either to relative estrogen deficiency (e.g., decreased recycling and enhanced fecal loss) or to relative estrogen excess (e.g., increased deconjugation and reabsorption), with downstream effects on estrogen-responsive tissues. The precise thresholds and configurations that distinguish a “healthy” from a “dysbiotic” estrobolome have not yet been defined, and the biological consequences of altered estrogen-metabolizing activity are likely to depend on the specific enzymes and substrates involved, as well as on host hormonal status and physiological context. Nonetheless, emerging evidence indicates that altered β-glucuronidase/sulfatase profiles are associated with estrogen-related disorders, including some hormone-dependent cancers, metabolic disturbances, and gynecologic pathologies such as endometriosis, endometrial hyperplasia, and possibly PCOS (21, 22). This suggests that estrobolome dysregulation may contribute to disease risk or progression by modulating local and systemic estrogen exposure.

## 4 Prebiotics and sweeteners

The exploration of alternative sweetening agents has long been driven by the need to mitigate the metabolic consequences of excessive sucrose consumption, such as obesity, type 2 diabetes, and cardiovascular diseases. However, modern nutritional science has shifted its focus from merely identifying inert sugar substitutes to discovering functional molecules that actively promote host health through the modulation of the gastrointestinal ecosystem. In this context, the integration of prebiotic functionality into sweetener design represents a major advancement in food science and molecular nutrition. By shifting the paradigm from chemical neutrality to metabolic activity, these compounds satisfy human sensory desires for sweetness while simultaneously serving as selective substrates for specific members of the colonic microbiota. Understanding the chemical and physiological mechanisms that allow a sweet molecule to act as a prebiotic requires a detailed analysis of its structural integrity during digestion, its interaction with taste receptors, its availability to the gut microbiota, its selective microbial utilization, including fermentation, and ultimately its effects on host health.

### 4.1 Definition and mechanisms of action of prebiotics

The current consensus definition of prebiotics was established by the International Scientific Association for Probiotics and Prebiotics (ISAPP): a prebiotic is formally classified as a substrate that is selectively utilized by host microorganisms conferring a health benefit (23). For a dietary substrate to exert a prebiotic effect through the colonic microbiota, it must successfully navigate a series of physiological steps within the human gastrointestinal tract. First, at least a fraction of the substance must escape complete digestion and absorption in the upper gastrointestinal tract and become available to the colonic microbiota. Second, upon entering the lower intestinal tract intact, the molecule must act as a specific metabolic substrate that is selectively utilized, including through fermentation, by target beneficial taxa, including but not limited to Bifidobacterium and Lactobacillus species, without promoting the overgrowth of co-existing pathobionts. Third, this selective microbial utilization must confer a demonstrated health benefit to the host, which may involve physiological, immunological, metabolic, or endocrine effects (Figure 2).

**Figure 2:**
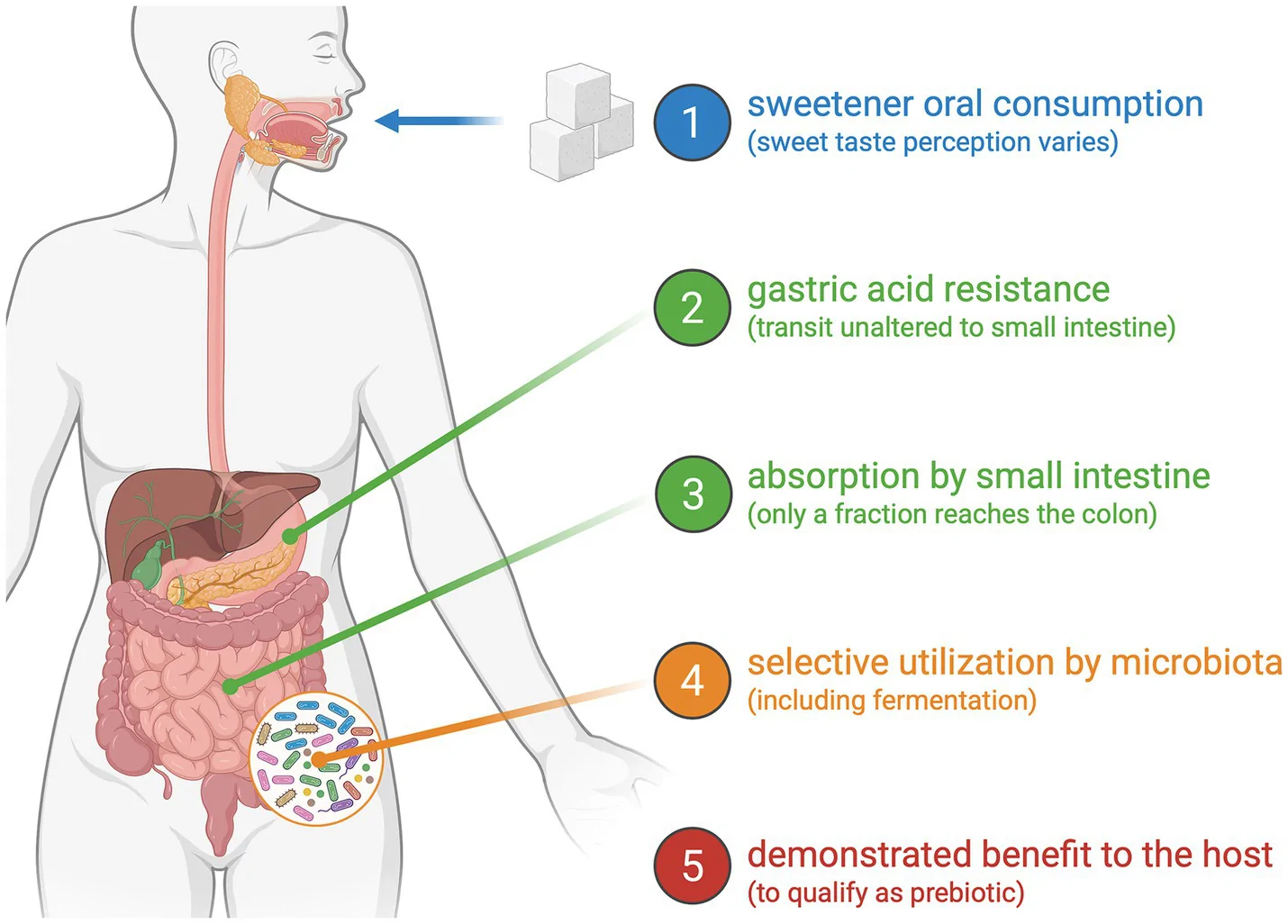
Characteristics of prebiotics. A prebiotic is defined as a substrate that is selectively utilized by host microorganisms, conferring a health benefit to the host. The figure illustrates gastrointestinal characteristics relevant to orally consumed substrates with potential prebiotic activity. Depending on their physicochemical and metabolic properties, these substrates may resist gastric conditions, undergo limited absorption in the small intestine, and consequently reach the colon to varying extents, where they may be selectively utilized by the gut microbiota, including through fermentation. These gastrointestinal features may influence substrate availability to intestinal microorganisms but do not constitute universal defining criteria for prebiotics. According to the consensus definition, qualification as a prebiotic requires selective utilization by host microorganisms and a demonstrated health benefit. Created in BioRender.

From a biochemical standpoint, the underlying mechanism of action relies entirely on the enzymatic machinery encoded within the metagenome of the resident saccharolytic microbiota. Beneficial bacteria express an array of specialized glycoside hydrolases that can cleave the complex, non-digestible glycosidic linkages characteristic of prebiotic molecules (24). This competitive fermentation pathway yields SCFAs, predominantly acetate, propionate, and butyrate, alongside gaseous byproducts such as carbon dioxide and hydrogen. The local accumulation of SCFAs drives down the luminal pH of the colon, establishing an acidic environment that may inhibit the growth of acid-sensitive pathogenic bacteria and enhance the solubility and absorption of certain minerals. Furthermore, SCFAs serve as energy substrates for colonocytes and function as signaling ligands that bind to host G-protein coupled receptors, contributing to the regulation of the tight-junction proteins, intestinal epithelial barrier and inflammatory responses (25–27).

### 4.2 When does a sweetener qualify as a prebiotic?

The functional intersection between sugar substitutes and prebiotic properties requires a single compound to fulfill two distinct biological criteria. As a sweetener, the compound must interact with oral sweet-taste receptors, primarily the T1R2/T1R3 heterodimer, while, to exert a prebiotic effect through the colonic microbiota, a sufficient fraction must escape digestion and absorption in the upper gastrointestinal tract and reach the colon. Finally, the fraction of the sweetener that reaches the colon must be selectively utilized by the resident microbiota, including, where applicable, through saccharolytic fermentation. Moreover, its effects on the microbiota should not result from broad-spectrum antimicrobial or bacteriostatic activity that indiscriminately disrupts the resident microbial community. Instead, its selective utilization should provide a metabolic advantage to beneficial microbial populations and may thereby contribute to the competitive exclusion of opportunistic pathogens (Figure 2).

### 4.3 Not all prebiotics are sweeteners, not all sweeteners are prebiotics

The paradigm of dietary formulation often conflates sensory attributes with metabolic outcomes, yet the biochemical intersection between prebiotics and sweeteners reveals that these two functions rely on entirely independent physiological pathways. To establish a dietary substrate as a prebiotic, reaching the lower gastrointestinal tract and undergoing selective microbial utilization, including through fermentation, are necessary but insufficient. The scientific consensus additionally requires that the substrate confers a demonstrated health benefits for the host. However, in contemporary nutritional literature, health benefits are sometimes inferred rather than directly demonstrated, as robust validation in controlled human studies remains limited for several candidate prebiotics, despite an accumulating but heterogeneous body of evidence (28). Conversely, the defining characteristic of a sweetener is strictly sensory. Crucially, the ability of a molecule to activate the T1R2/T1R3 sweet-taste receptor does not determine its ability to resist host enzymatic cleavage, escape absorption in the small intestinal transit, or effectively reach the colon. Consequently, the potential dual role of a dietary carbohydrate or sugar substitute as both a sweetener and a prebiotic can be evaluated according to three key physiological characteristics. First, the substance must be characterized for its sweetening potency relative to sucrose, establishing its functional viability as a sugar alternative. Second, the proportion of the ingested dose that escapes upper gastrointestinal absorption and reaches the colon must be estimated. Third, researchers must verify whether this colonic fraction is selectively utilized by the resident intestinal microbiota, including through fermentation. If a sweetener successfully navigates this pathway and undergoes selective microbial utilization in the colon, it qualifies as a prebiotic only when this is accompanied by a demonstrated health benefit for the host. In the context of modern microbiome research, this beneficial effect may be reflected by the selective expansion of well-established beneficial taxa, such as Bifidobacterium or Lactobacillus (28).

However, the selective proliferation of specific microbial taxa is not universally beneficial and may, depending on the taxa involved, have adverse consequences for the host. If a sweetener selectively promotes pathogenic or potentially harmful taxa rather than beneficial ones, the resulting physiological effects may be detrimental to the host. A potential example relevant to the present review is the enrichment of bacterial populations harboring high-activity β-glucuronidase enzymes. As a functional component of the estrobolome, increased microbial GUS activity promotes the deconjugation of glucuronidated estrogens in the gut lumen. This enzymatic deconjugation allows free estrogens to be reabsorbed into the portal circulation through enterohepatic recirculation, potentially increasing systemic estrogen exposure and influencing estrogen-responsive tissues (Figure 1) (7–10). Therefore, a comprehensive overview of these divergent physicochemical and physiological characteristics is important for evaluating the potential interactions between sweeteners, prebiotic properties, and the gut microbiota, as illustrated in Table 1, which compares the sweetness potencies, colonic availabilities, and fermentation characteristics of major sweeteners and prebiotic compounds.

## 5 Sweeteners as prebiotics: functional modulation of the gut microbiome and estrobolome

### 5.1 Sweeteners as prebiotics for the gut microbiome

Animal studies and some clinical data in humans indicate that several artificial sweeteners can modify gut microbiota composition and function in ways that may have metabolic consequences (29). Saccharin and sucralose, in particular, have been associated in rodent models with reduced microbial diversity, shifts in the Firmicutes/Bacteroidetes balance, and enrichment of certain Proteobacteria, sometimes in parallel with impaired glucose tolerance, increased adiposity, and low-grade inflammation (30–33). Similar findings have been reported for acesulfame-K (34) and, in some settings, for mixtures of non-caloric sweeteners, although results are not always consistent across strains, doses, and experimental designs. Human studies are fewer and more heterogeneous but suggest substantial inter-individual variability. In controlled trials, some individuals exhibit microbiota-dependent glycemic responses to non-caloric sweeteners, with specific baseline microbial configurations associated with differential responses, ranging from worsened glucose tolerance to largely neutral effects (35). In other cohorts, modest compositional changes have been observed, such as alterations in Bacteroides, Ruminococcus, or specific Clostridia, without clear clinical consequences, highlighting the complexity of extrapolating from short-term interventions to long-term health effects (36).

Several mechanisms have been proposed to explain how artificial sweeteners might influence the gut microbiome. These include selective pressure favoring bacteria that can tolerate, accumulate, or transform these xenobiotics; indirect effects via changes in luminal osmolarity, pH, or bile acid composition; and activation of sweet-taste receptors (T1R2/T1R3) within the gastrointestinal tract, modulating motility, enteroendocrine signaling, and mucus secretion, thereby altering the habitat for resident microbes (37). In addition, some artificial sweeteners may interact with regulatory pathways controlling microbial gene expression, including those involved in carbohydrate metabolism and stress responses (37). Overall, the pattern and magnitude of microbiome alterations induced by artificial sweeteners in habitual human consumers, especially at doses and durations that reflect real-world intake, remain incompletely defined. Most data derive from animal studies using relatively high doses or from short-term human interventions in small samples.

Caloric sweeteners, particularly when consumed in amounts exceeding small-intestinal absorptive capacity, can affect the microbiome by increasing the amount of carbohydrate that reaches the colon. This additional substrate load can shift the balance of microbial metabolism toward saccharolytic or, when coupled with high fat and low fiber, more proteolytic fermentation, with downstream effects on luminal pH, SCFA production, gas emission, and generation of potentially harmful metabolites (e.g., branched-chain fatty acids, certain phenols and amines). Diets characterized by high intake of sugars are also linked to low-grade metabolic inflammation and changes in bile acid composition that can further remodel the gut ecosystem, favoring taxa adapted to a high-energy, low-fiber niche (37).

Polyols such as xylitol and sorbitol are only partially absorbed in the small intestine and are readily fermented by colonic microbes. Their presence in the colon increases osmotic load and gas production and, in some individuals, can cause bloating or diarrhea. Fermentation of these polyols can generate SCFAs and select for taxa that efficiently utilize these substrates, including specific Bacteroides and Firmicutes lineages. Over time, this may modestly re-shape community composition toward organisms specialized in polyol metabolism, although the net health impact likely depends on overall diet and dose (38, 39).

Steviol glycosides and other plant-derived high-intensity sweeteners are not absorbed intact. Steviol glycosides, for example, require microbial deconjugation in the colon to release steviol, which is then absorbed and further conjugated in the liver. This processing transiently engages specific bacterial groups with the requisite glycosidase activities, but the total mass of substrate is small, and current human and animal data suggest only modest effects on overall community structure at realistic consumption levels. Similar considerations may apply to other natural high-intensity sweeteners that interact with the gut microbiota, although current evidence does not suggest major shifts in overall gut microbiota composition at realistic consumption levels (40, 41).

### 5.2 Sweeteners as prebiotics for the estrobolome?

To authors’ knowledge, no trials have yet been designed with β-glucuronidase/sulfatase activity and estrogen kinetics as primary outcomes. However, potential effects of sweeteners on the estrobolome can be inferred from their reported effects on the gut microbiome and on specific estrobolome-relevant taxa. Given their documented effects on overall microbiota composition and function in some animal and human models, it is plausible that high or chronic intake could shift the abundance of taxa harboring estrobolome-related genes and potentially alter the expression or activity of the encoded enzymes. For example, increases in Proteobacteria or specific Bacteroides and Clostridium species, groups known to include high-activity β-glucuronidase producers, might alter the net deconjugation capacity within the gut and, consequently, the balance between estrogen excretion and recirculation. Conversely, loss of microbial taxa contributing to GUS/SULF activity could potentially reduce the overall deconjugation capacity of the estrobolome, depending on the expression and activity of the relevant estrogen-metabolizing genes and enzymes within the affected taxa. In addition to microbiota compositional effects, some non-caloric sweeteners may interact with microbial xenobiotic-sensing systems, stress-response regulators, and global transcriptional networks, potentially up- or down-regulating expression of conjugating/deconjugating enzymes independently of gross taxonomic shifts (21, 42, 43). Changes in luminal pH, osmolarity, and bile acid pools induced by chronic sweetener exposure might further modulate enzyme expression and activity. From the host perspective, altered microbial metabolites (e.g., SCFAs, secondary bile acids) could influence hepatic conjugation pathways and enterohepatic cycling, indirectly impacting the substrate load available to the estrobolome. At present, these remain largely speculative considerations, inferred from broader microbiome data rather than estrobolome-focused measurements.

Sugars and other fermentable sweetening compounds may influence the estrobolome primarily by reshaping the overall gut microbial ecosystem rather than by acting as direct substrates for estrogen-modifying enzymes. Diets rich in refined sugars and sweetened foods can promote expansion of saccharolytic and, in some contexts, proteolytic taxa, reduce microbial diversity, and favor pro-inflammatory, bile-acid–modifying communities. These shifts could indirectly alter the abundance of bacteria carrying β-glucuronidase and sulfatase genes and, if accompanied by corresponding changes in gene expression or enzymatic activity, could potentially affect the net deconjugation capacity of the estrobolome (44).

Chronic high-sugar, low-fiber dietary patterns are also associated with metabolic inflammation, insulin resistance, and altered enterohepatic circulation of bile acids, all of which can feed back on microbial composition and enzyme expression, further complicating the relationship between sugar intake and estrogen metabolism.

Fermentable polyols and certain plant-derived compounds (e.g., oligosaccharides co-present with natural sweeteners) may selectively enrich or deplete specific taxa harboring β-glucuronidase/sulfatase genes. Polyols such as xylitol and sorbitol, by serving as colonic substrates, can favor growth of bacteria adapted to their metabolism, some of which may possess estrobolome-related genes. High-intensity plant sweeteners like steviol glycosides require microbial deconjugation, engaging specific glycosidase-producing bacteria, but the quantities involved and current data suggest only modest effects on the broader gut microbial community, with any corresponding effects on the estrobolome remaining inferential (39).

### 5.3 Comparing sweeteners for gut microbiome and estrobolome interactions

Common sweetener candidates differ in their potential effects on the gut microbiota and, by extension, on the estrobolome. Fermentable polyols such as xylitol, sorbitol and maltitol reach the colon in substantial amounts and are readily fermented by bacterial groups that can alter fermentative profiles, increase gas production, and select for taxa with potential β-glucuronidase or sulfatase activity; with high or prolonged intake, these changes may therefore influence estrobolome composition and function. Lactitol behaves similarly as a highly fermentable disaccharide polyol with approximately 90 % colonic availability; its metabolism by saccharolytic microbes significantly alters the luminal environment by lowering the pH and stimulating butyrate production, while potentially selecting for bacterial taxa that include high-activity GUS producers. Some non-nutritive sweeteners (e.g., sucralose, saccharin, acesulfame-K) have been associated in animal models with reduced microbial diversity, shifts in the Firmicutes/Bacteroidetes ratio and enrichment of Proteobacteria, although human data are heterogeneous and context-dependent; such alterations could indirectly affect estrobolome function if they translate into changes in the expression or activity of estrogen-metabolizing enzymes. Polydextrose is another sweetener that readily reaches the colon because it is poorly digested in the small intestine and is subsequently fermented by the resident microbiota (45). Accordingly, polydextrose acts as a low-calorie bulking agent with recognized prebiotic properties: its microbial utilization promotes SCFA production and can enrich specific fermentative taxa. Its colonic availability therefore can alter microbial community structure and metabolic outputs and, depending on the functional characteristics of the affected taxa and the expression and activity of relevant estrogen-metabolizing enzymes, may potentially influence estrobolome function (46). Plant-derived high-intensity sweeteners (e.g., steviol glycosides, mogrosides) can interact with specific bacterial groups during their colonic metabolism, but the substrate load is generally small and effects on overall microbiota composition appear modest at realistic consumption levels, albeit not entirely absent. In short, many sugar substitutes are not microbiota-neutral: they differ in colonic fermentability, can exert selective pressure on particular taxa, or may have xenobiotic effects that, especially with chronic use, could influence estrobolome composition and function.

Among the sweeteners considered, a distinct profile is observed for erythritol, whose limited colonic availability and poor fermentability are expected to minimize its direct interaction with the intestinal microbiota. Erythritol is rapidly and efficiently absorbed in the small intestine and is largely excreted unchanged in the urine, so only a very small fraction reaches the colon. Unlike most polyols, it is poorly fermented by colonic bacteria and therefore provides minimal substrate for colonic microbial growth or metabolic reshaping (Figure 3). Erythritol also provides sweetness with negligible caloric contribution and generally good gastrointestinal tolerability at typical doses compared with other fermentable polyols, while its organoleptic profile and technological stability make it a practical sucrose substitute in many applications.

**Figure 3:**
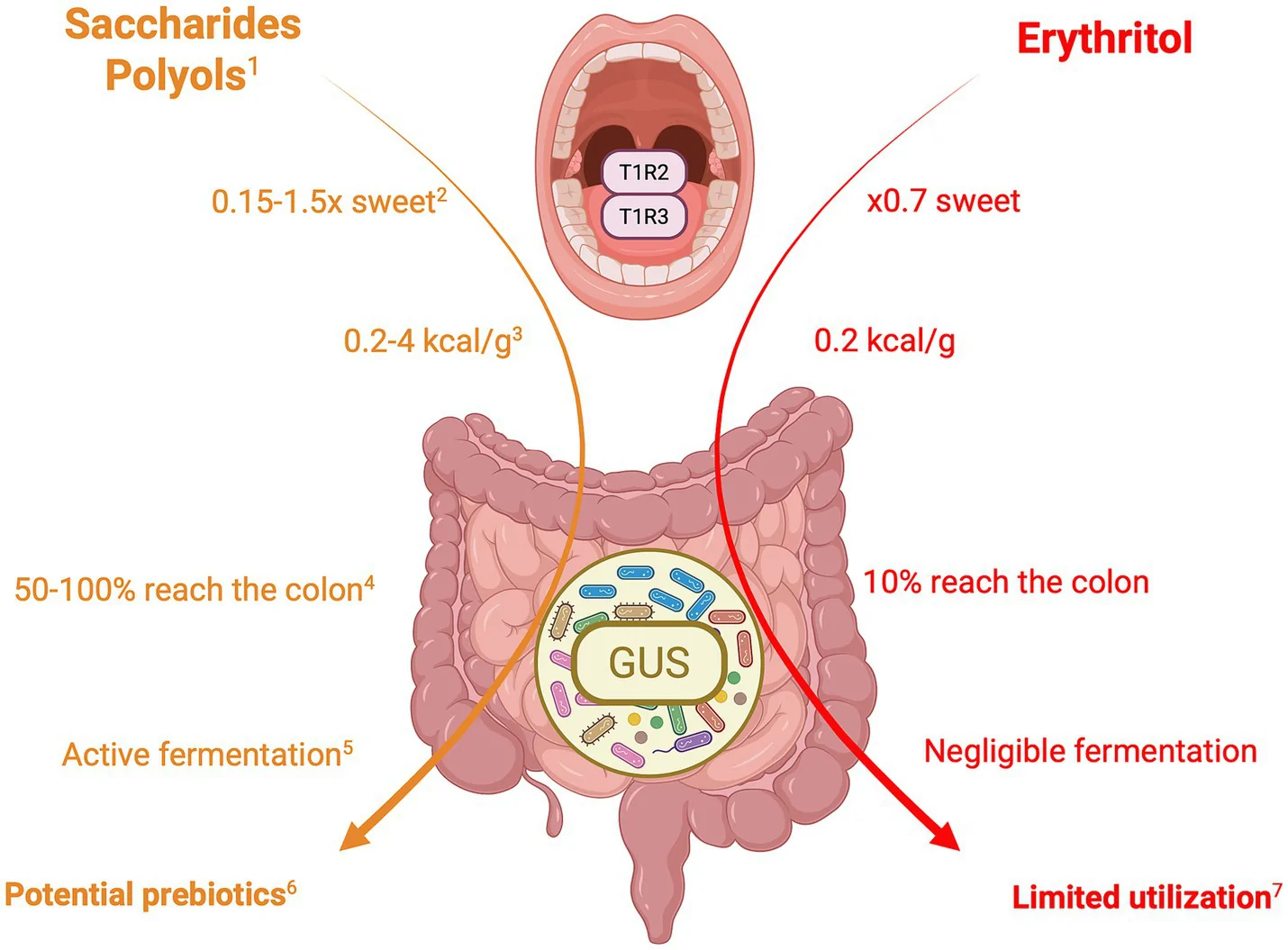
Comparison between erythritol and other polyols or saccharides. Erythritol exhibits a distinctive metabolic profile, as only a small proportion reaches the colon and its fermentation by the gut microbiota is negligible, resulting in limited availability for microbial utilization. In contrast, several other polyols and saccharides reach the colon in larger amounts and undergo active microbial fermentation. Such fermentation may contribute to prebiotic activity when selective microbial utilization is associated with a demonstrated health benefit; however, fermentation per se does not imply either beneficial or detrimental effects, which depend on the substrate and dose, host and microbial context, microbial functions, and resulting metabolites. β-Glucuronidase (GUS); ^1^polyols excluding erythritol; ^2^except sucralose (~600×); ^3^except sucralose (0 kcal/g); ^4^except sucrose and monosaccharides (0–30%); ^5^except glucose, sucrose and sucralose; ^6^potential prebiotic activity requires selective microbial utilization associated with a demonstrated health benefit; fermentation alone does not establish a prebiotic effect; ^7^limited utilization is inferred from the low proportion reaching the colon and negligible fermentation and does not imply microbiome or estrobolome neutrality or exclude indirect effects. Created in BioRender.

Because erythritol reaches the colon only in small amounts and is poorly fermented (47), it would be expected to exert limited direct selective pressure on bacterial taxa harboring β-glucuronidase and sulfatase genes and, consequently, to have less potential than fermentable polyols or certain artificial sweeteners to enrich or deplete estrobolome-relevant taxa or alter microbial enzymatic activity involved in enterohepatic estrogen recirculation (48). Practical caveats remain although mechanistic and short-term human data support a limited impact of erythritol on microbiota composition, direct evidence linking erythritol intake to changes in estrobolome function or estrogen kinetics in humans is lacking. Until longitudinal, functionally oriented studies are available, interpretation of these findings should remain cautious. In summary, among commonly used non-caloric sweeteners, erythritol presents a distinctive profile characterized by high small-intestinal absorption, low colonic availability, and poor fermentability, which together are expected to limit its direct interaction with the gut microbiota and, by inference, its potential effects on the estrobolome.

Nonetheless, the potential effects of a sweetener on the microbiome should be considered within the context of its overall health effects. Erythritol has been extensively studied *in vivo* and, to a lesser extent, in clinical settings. Although erythritol was initially considered a potentially beneficial sweetener for patients with diabetes because of antioxidant properties that might protect the endothelium from hyperglycemia-induced vascular damage (49–51), concern has arisen in recent years. *In vitro* and preliminary clinical studies have associated erythritol with increased platelet activation, raising concerns about thrombosis and potential cardiovascular and cerebrovascular events (52–55). However, because many studies are *in vitro* and the clinical data are preliminary and have been criticized for possible confounding, these findings should be interpreted with caution (56, 57). Further clinical studies are warranted to determine the clinical significance and potential causality of these associations.

## 6 Research gaps and future perspectives

Major gaps remain in our understanding of how sweeteners influence the estrobolome. First, causal evidence is scarce: few to no studies have simultaneously measured sweetener intake, microbiota composition, estrobolome gene/enzyme activity, and estrogen metabolites in well-phenotyped cohorts. Consequently, the successive links between sweetener exposure, microbial composition and function, estrogen-deconjugating enzyme activity, and estrogen metabolism remain largely inferential and require direct experimental validation. Second, dose–response relationships and the impact of realistic, long-term consumption patterns are poorly defined, especially in women at different life stages and hormonal states (e.g., premenopause, pregnancy, menopause, endocrine therapy). Third, we lack detailed information on which specific β-glucuronidase and sulfatase families are most responsive to dietary sweeteners and how their modulation translates into clinically meaningful changes in estrogen exposure. Future research should prioritize controlled feeding trials and longitudinal cohort studies integrating metagenomics, metatranscriptomics, direct assessment of β-glucuronidase and sulfatase activities, and metabolomic profiling of both free and conjugated estrogens, together with clinical endpoints relevant to women’s health (Figure 4).

**Figure 4:**
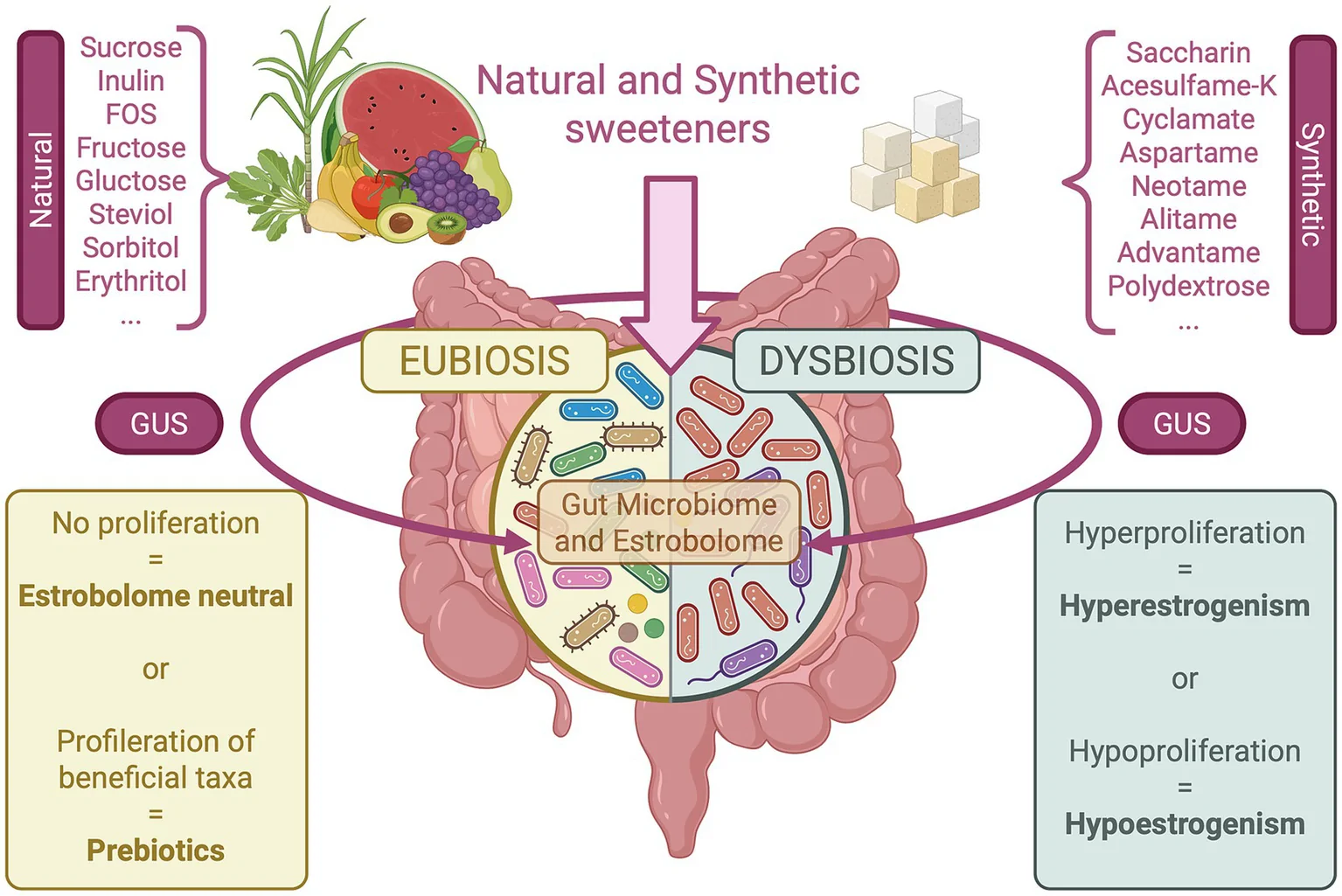
Conceptual representation of the potential relationships between different sweeteners, gut microbiome composition, and the estrobolome. Sweeteners are grouped as natural or synthetic and their potential effects are represented in relation to eubiosis or dysbiosis, microbial proliferation, prebiotic activity, estrobolome neutrality, and possible changes in estrogen status.

## 7 Discussion and conclusion

Sweeteners are defined by their capacity to provide sweetness, but they differ in their intestinal absorption, colonic availability, and susceptibility to microbial transformation. This heterogeneity shapes the routes through which each compound may interact with the gut microbiota, so findings for one sweetener cannot be generalized uncritically to the category as a whole. Some individual sweeteners have been associated with changes in gut microbial composition and metabolic outputs in experimental models and human studies, although the direction and magnitude of these associations vary with compound, dose, exposure duration, study model, and host context (31, 58). Microbial β-glucuronidase and sulfatase activities, in turn, contribute to the deconjugation of estrogen conjugates excreted in bile and thereby to enterohepatic estrogen recycling, linking the gut microbiota to systemic estrogen availability (13, 14, 59). A sweetener-associated microbial response could therefore include changes in microbial estrogen metabolism, although changes in the abundance of taxa known or presumed to harbor relevant genes cannot establish that connection (59). Taxon abundance does not show which strains are present, whether they carry and express the relevant genes, or whether the corresponding enzymes act on estrogen conjugates. The unresolved functional question is whether exposure to a defined sweetener is associated with changes in estrogen-relevant gene expression or enzyme activity. The physiological question is whether any such change is accompanied by altered systemic profiles of conjugated and unconjugated estrogens.

That functional question cannot be separated from the intestinal fate of each compound. Colonic availability provides the opportunity for direct luminal interaction, but it cannot by itself determine whether a microbial effect occurs because proposed mechanisms also include gastrointestinal signaling or changes in the intestinal habitat (37). Prebiotic classification answers a separate question: selective microbial utilization must be accompanied by a demonstrated health benefit in the target host (23). Fermentation or enrichment of selected taxa alone does not meet that standard, and prebiotic classification does not predict whether estrogen deconjugation changes, the direction of any change, or its clinical significance. Estrobolome alterations have been associated with estrogen-related conditions (7–10, 21, 22), but those associations do not establish that higher or lower β-glucuronidase or sulfatase activity is intrinsically beneficial or harmful. Erythritol makes this inferential boundary concrete. Its high small-intestinal absorption, low colonic availability, and poor fermentability together suggest that the opportunity for direct microbial interaction is comparatively limited (47, 48), but this profile does not demonstrate an absence of effects on microbiota composition, microbial function, or estrobolome activity. It also does not resolve the separate cardiovascular safety signals, which remain preliminary and include potentially confounded associations (52–57). Intestinal disposition can therefore help identify plausible routes of interaction and guide compound-specific testing, while judgments of benefit or harm remain tied to the relevant outcome evidence.

Concern about obesity, diabetes, and cardiometabolic disease has contributed to a food-system shift from conventional sugars toward alternative sweeteners (1). These compounds share sweetness but not a common biological fate. The metabolic outcomes that motivated this substitution remain essential to its assessment, alongside evidence on safety and tolerability. The shift toward One Health places those intended outcomes and the potential biological consequences of the resulting exposure within the same scientific question. For sweeteners, the assessment therefore extends from compound-specific intestinal disposition and microbial function to any demonstrable consequence for host endocrine physiology. Within microbial endocrinology, that question becomes testable by jointly evaluating a defined exposure, estrogen-relevant GUS and SULF activities, and systemic profiles of conjugated and unconjugated estrogens in a clearly characterized hormonal and clinical context. Clinical decisions should remain grounded in the available metabolic and safety evidence, while the untested sweetener–estrobolome link remains an explicit uncertainty rather than being interpreted as endocrine neutrality. Because the food-system transition is already under way, scientific assessment must accompany it by testing for biological effects at the level of the individual compound, the relevant microbial function, and the host context.
